# Molecular biomarkers of progression from Barrett’s esophagus to esophageal adenocarcinoma

**DOI:** 10.3389/fgstr.2023.1007456

**Published:** 2023-05-25

**Authors:** Luke Taylor, Hani Naeem Alastal, Ashraf Rasheed

**Affiliations:** ^1^ Department of Oesophago-Gastric Surgery, University Hospital Bristol and Weston NHS Foundation Trust, Bristol, United Kingdom; ^2^ Department of Translational Medicine, University of South Wales, Newport, United Kingdom; ^3^ Department of Upper Gastrointestinal Surgery, Royal Gwent Hospital, Aneurin Bevan University Health Board, Newport, United Kingdom

**Keywords:** biomarker, Barrett’s oesophagus, dysplasia, oesophageal adenocarcinoma, cyclin D1, β-catenin, p53

## Abstract

**Introduction:**

Barrett’s esophagus (BO) is a pre-malignant condition for esophageal adenocarcinoma (OAC), the incidence rate of which has risen dramatically over the last four decades in the Western world. The 5-year survival rate of OAC is poor, and one of the ways to improve it would be by focusing on identifying high-risk Barrett’s patients through a surveillance program. Currently, histologic dysplasia is the only recognized marker of progression to OAC. Molecular biomarkers found in tissue samples that predict which patients have a higher risk of progression to OAC may act as a reliable tool for the stratification of patients with BO.

**Aim:**

To determine whether molecular biomarkers have a potential use in predicting which patients with BO have a higher risk of progression to OAC.

**Methods:**

Immunohistochemistry was performed on 25 tissue samples obtained from the endoscopic biopsies of 19 patients with confirmed BO. Hematoxylin and eosin (H&E) staining was used to confirm the presence of BO and dysplasia. Staining was performed in an external independent laboratory. Statistical analysis using the Mann–Whitney *U* test was performed using R Studio^®^ statistical software.

**Results:**

Of the 19 patients sampled, three had low-grade dysplasia (LGD), and all had confirmed metaplasia diagnostic of BO. Expression of cyclin D1 was noted to be elevated in patients with LGD compared with those with metaplasia only (*p* = 0.042). Expression of Sox2 was elevated in metaplastic BO cells compared with normal squamous cells within the same stain (p = 0.046). Of all eight biomarkers tested, β-catenin had the greatest overall expression (p < 0.004).

**Conclusions:**

Isolating elevated cyclin D1 in patients with LGD highlights its potential use as a biomarker in identifying BO patients at risk of developing dysplasia, and, in turn, their possible progression to OAC. Elevated levels of both Sox2 and β-catenin may also serve as markers for disease progression when overexpressed in BO patients. Both conclusions, however, would need long-term follow-up to fully establish their prognostic usefulness, as at the time of writing no patients in this study had gone on to develop OAC. Although only a small sample size was present for this study, and follow-up was limited, it serves as a strong pilot for further research into the use of novel biomarkers in predicting which BO patients are at high risk of developing dysplasia and progressing to OAC.

## Introduction

The global incidence of esophageal adenocarcinoma (OAC) is increasing, with around 9,300 new cases recorded per year in the UK (1). The 5-year survival rate of OAC is currently 17%, making it the seventh commonest cause of cancer-related death in the UK (1). With the incredibly poor prognosis of OAC it is essential to diagnose and treat it early, with increasing importance being placed on the diagnosis and treatment of its precursor, Barrett’s esophagus (BO) (2). BO is a pre-malignant condition defined as the presence of metaplastic columnar epithelium within the distal esophagus, containing goblet cells on histology (3). These metaplastic epithelia can undergo further changes into low- and high-grade dysplasia (4), with high-grade dysplasia offering the greatest risk of malignant transformation (5). The presence of dysplasia increases the risk of progression to OAC from 0.33% to 1.40% (6), and high-grade dysplasia leads to a 40-fold increased risk of developing OAC in the general population (7).

The current gold standard of treatment is regular endoscopic surveillance of BO with quadratic biopsies to monitor for signs of dysplasia and for progression to OAC (8). Currently, there is no marker or predictor for patients at higher risk of developing high-grade dysplasia or OAC; therefore, all patients are offered regular surveillance and biopsies (9). However, despite regular surveillance programs, the rates of OAC continue to rise (10, 11). With the new role of endoscopic submucosal resection there is an increased clinical need to identify and prioritize high-risk individuals who will benefit from endoscopy and submucosal resection (2, 8), which is a limited resource (12, 13). A variety of biomarkers that have been associated with esophageal cancer have been proposed to aid in this risk stratification (9, 14). Earlier identification of high-risk patients by using a biomarker panel may help play a role in providing earlier treatment and improving 5-year survival rates.

Extensive literature and a meta-analysis performed by Alastal (15) have highlighted multiple biomarkers to be further trialed and tested within the BO population. These markers include P53, cyclin D1, and P16, as well as several other markers that had been previously analyzed in stage 3 studies (cyclin A, Sox2, Cox2, β-catenin, and Ki67) (16–20). Biomarkers can play a role as both diagnostic and prognostic markers, and increasing evidence suggests that a panel of markers, rather than a single marker, offers far more accuracy and predictability in its results (21). Adjunct markers to the current histologic assessment may prove useful in helping to predict dysplastic and further metastatic changes in BO cells, helping to stratify and quantify the need for ongoing surveillance frequency and the need for more invasive treatment (22).

The overexpression of P53 is already established within many cancers and has recently been used alongside a protein signature to predict progressive disease in BO (9). Overexpression of cyclin D1 has also been established within the proliferation of cancer cells, playing a large role within the cell cycle (23). We highlight the role of cyclin D1 as a potential marker of dysplasia (i.e., progression of disease), which may also offer prognostic factors, as seen with forms of breast cancer (24).

The overall aim of this study was to identify biomarkers that may play diagnostic and prognostic roles for patients with BO, and to highlight where further research is required in the creation of such a biomarker panel to identify patients with BO who are at a higher risk of disease progression.

## Methods

### Patient groups

Patients undergoing endoscopic surveillance for BO on the Royal Gwent Hospital (RGH) register were selected for inclusion in the study. Full inclusion and exclusion criteria are shown in **Table 1**. Ethical approval was obtained from the Welsh Research Ethics Committee. Patients consented to have their biopsied tissue samples included in the study. Based on previously published studies (19), the sample size for an unmatched case–control study was calculated using the Kelsey method, in order to detect a statistically significant effect size, with an 80% power (25).

**Table 1:** showing inclusion and exclusion criteria for patients included within the study.

Inclusion criteria	Exclusion criteria
Patients who had histologically proven BO with at least one baseline endoscopy	Patients on warfarin or those with bleeding disorders
Patients with BO including low- and high-grade dysplasia	Patients who had received chemotherapy
Patients aged > 18 years	

### Tissue samples

Tissue samples for immunohistochemistry (IHC) analysis were acquired from the endoscopic biopsies of 19 BO patients at the RGH. Sections of each tissue sample were cut to 4 µm and mounted on histology slides to be used for IHC analysis. Slides were first stained with haemotoxylin and eosin and reviewed by an independent consultant pathologist to determine the histologic grade according to the Vienna classification (26). The same independent consultant pathologist then identifed the sample with the most abnormal finding for testing the biomarkers using IHC analysis. If dysplasia was not found, then a random sample was selected for the marker staining.

### Immunohistochemistry

Immunohistochemistry staining for cyclin D1, P53, P16, cyclin A, Sox2, Cox2, β-catenin, and Ki67 was performed on 25 single slides of 4-µm-thick tissue sections cut from formalin-fixed paraffin-embedded blocks of tissue samples. Staining was performed by Professor Manuel Rodriguez-Justo at an independent laboratory, and the expression of each marker was assessed as a percentage of the stain. All marker expressions were scored based on a scale of 0 to 3, with 0 being negative for staining and 3 being strongly positive with full thickness cover. The methods used have been previously validated and published in the literature by Bird-Liberman et al. (19), with techniques for staining, images, and scoring available for open access review.

### Statistical analysis

The percentage of marker expression was matched to the presence of metaplasia, low-grade dysplasia, and high-grade dysplasia. Mann-Whitney *U*-testing was performed to compare the percentage expression with the presence of metaplasia vs. dysplasia. Two-way ANOVA testing was performed to compare the percentage expression of the different markers. All statistical analyses were performed using R studio^®^ statistical software and figures were generated using GraphPad^®^ Prism 9 software.

## Results

### Analysis of patient cohort and expression of markers

**Figure 1:**
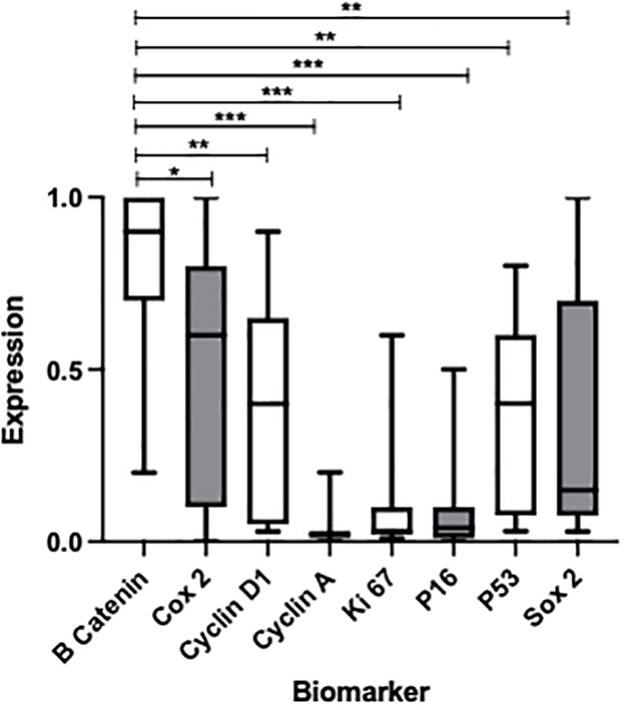
Box plot to show the expression of multiple biomarkers after H&E staining of metaplastic BO tissue samples obtained from endoscopic biopsy. P-values represent comparison of β-catenin, showing the greatest overall expression, with other biomarkers *p < 0.0472, **p < 0.0037, ***p < 0.001. Median percentage expressions of each marker and interquartile ranges are as follows from left to right: 2 (1–3), 15 (1–70), 60 (10–80), 90 (70–100), 40 (10–60), 4 (1–10), 3 (2–10), and 40 (5–60).

In total, 19 patients were included in the study providing endoscopic biopsies, with the final analysis being performed on a total of 25 slides of tissue samples. Full patient demographics, with histology, and number of patients progressing to cancer, are shown in **Table 2**. Three of the patients had evidence of dysplasia, with none having adenocarcinoma. IHC analyses were performed using a total of eight potential biomarkers, with the full analysis between each marker being shown in **Figure 1**.

**Table 2:** Demographics of all 19 patients, including their histologic changes and progression. Values given are medians with upper and lower interquartile ranges.

Age (years)	74 (68–78)
**Male**	n = 14
**Female**	n = 5
**BMI**	27 (25–34)
**Metaplasia**	
** Yes**	17
** No**	2
**Dysplasia**	
** Yes**	5
** No**	14
**Cancer progression within one year**	
** Yes**	1
** No**	18

β-catenin was noted to show the greatest expression in BO cells when compared with the expression noted of any other marker (p<0.0472; see **Figure 1** for full p-values). Significant elevations of expression were also noted of Cox2 and cyclin D1, as well as P53 and Sox2 to a lesser degree. However, these values were not statistically significant across the board; elevations of expression were apparent only when compared with the markers showing the lowest expression (cyclin A, Ki 67, and P16).

### Use of markers to identify the presence of dysplasia

**Figure 2:**
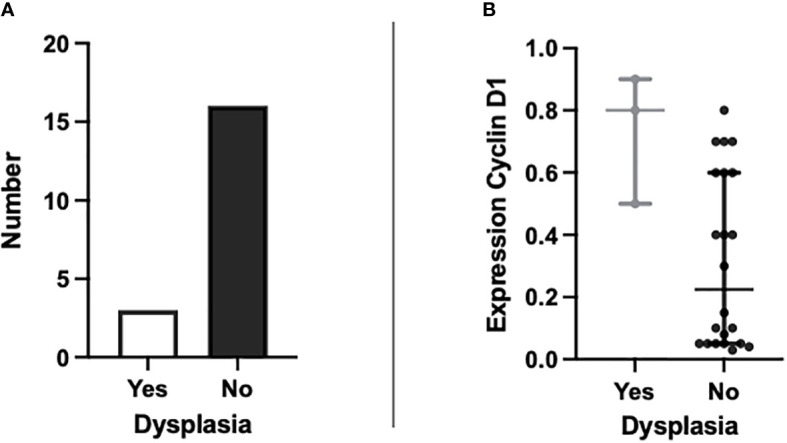
Graphs to show **(A)** the number of patients with BO showing evidence of dysplasia present on H&E staining (n=3) and those without dysplasia (n=16); and **(B)** the percentage expression of cyclin D1 in patients with dysplasia present on H&E staining against patients without dysplasia (p = 0.042).

Of the 19 patients providing tissue samples, three had evidence of dysplasia, which is shown in **Figure 2A**. Comparison analyses were performed with all eight markers for differences in expression between metaplastic BO cells and dysplastic BO cells. Cyclin D1 showed a significant elevation in expression between dysplastic and non-dysplastic cells (p 0.042, see **Figure 2B** **)**. The increased expression of cyclin D1 appears to be indicative of dysplasia from simple metaplastic BO and therefore may act as a potential marker for use in the differentiation of low- to high-risk patients.

### Need for control variables and the potential for diagnostic biomarkers

**Figure 3:**
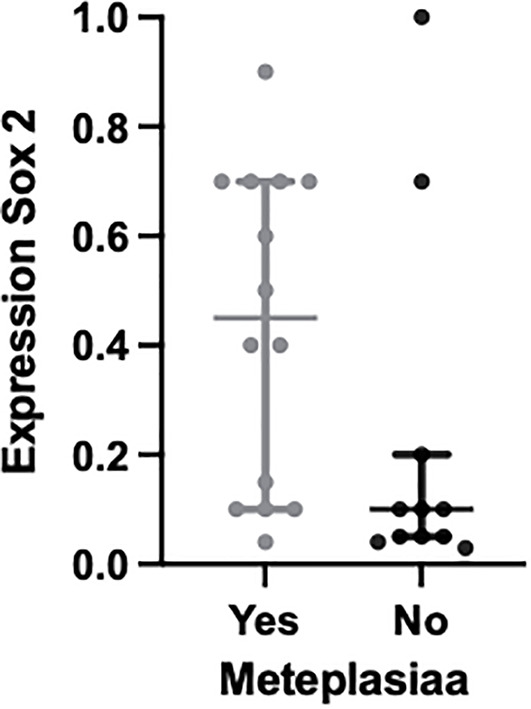
Plot to show the expression of the molecular biomarker Sox2 in metaplastic BO cells compared with normal squamous cells within the same tissue samples; p = 0.046.

As seen in **Figure 1**, we note that multiple markers are elevated in patients with BO. With promising data in the use of markers to highlight the presence of dysplasia from metaplasia, the markers were also analyzed to show whether they had an increased expression between normal esophageal squamous cells and metaplastic BO cells within the same tissue samples. The percentage of expression of Sox2 was seen to be elevated in metaplastic cells compared with normal squamous mucosa (p = 0.046), offering a possibility of a diagnostic biomarker (**Figure 3** **)**. This result is limited, however, as no control variables existed in this patient cohort: normal esophageal squamous cells were present only where these cells happened to be biopsied along with the Barrett’s cells during endoscopy.

## Discussion

Use of biomarkers within the field of Barrett’s esophagus and esophageal adenocarcinoma is without doubt a continually developing field, with a growing need for the development of a panel to identify disease progression. A multitude of biomarkers show the potential to offer a variety of information with regards to identification of disease, progression of disease, and prognosis of disease (27). With regards to improving patient outcomes and treatment availability, the use of diagnostic markers, and, more importantly, progression markers, will play the greatest role in increasing the 5-year survival rates for patients (28). Current risk progression from BO to OAC is estimated to be approximately 0.5% per patient (29, 30). This relatively low risk of progression in what is a relatively large proportion of the population reinforces the need for a tool to stratify the most at-risk patients for more intense surveillance and earlier treatment intervention.

Highlighting patients who are at risk of disease progression with a single biomarker or a panel of biomarkers will isolate an at-risk population for whom limited screening resources are available (31). Biomarkers such as MCM2 (minichromosomal maintenance 2) expression and loss of heterozygosity (loss of normal function of one allele within a gene with an already inactive second allele) have shown the most promise (32, 33). However, the cost of these markers and laboratory time needed limit their use in clinical practice (27). IHC analysis offers a cheaper and less time-constrictive method for applying biomarkers, for which cyclin D1, cyclin A, and P53 have shown their potential to identify BO patients with the highest risk progression to OAC (17, 34–36). Our study shows that cyclin D1 is overexpressed in dysplastic BO cells compared with standard metaplastic BO cells (p = 0.042), suggesting that a higher expression of cyclin D1 is a marker for disease progression to dysplasia. What is left to be concluded, however, is whether cyclin D1 therefore acts as a marker for further disease progression from dysplasia to OAC. If cyclin D1, or a combination of the above markers, can be used within a receiver operator characteristic to predict further disease progression, then their early overexpression can select a target population for more intensive surveillance and early treatment with either endoscopic ablation or surgical intervention (37).

Cyclin D1 has been described in the literature as an important regulator of the cell cycle phases G1 to S phase and is a critical proto-oncogene in the regulation of cell cycle progression (38). Overexpression of cyclin D1 is therefore linked to the development and progression of cancer (38). Other studies have described cyclin D1 as a prognosticator for confirmed OAC due to the increased genomic instability associated with the overexpression of cyclin D1 (39, 40). Our results, along with the existing literature, prompt further research into the use of cyclin D1 both in predicting progression and in the prognosis of confirmed cancer. This would involve prospective research with IHC analysis used to measure the expression of cyclin D1 in metaplastic BO, dysplastic BO (including high- and low-grade dysplasia cells), and OAC, with follow-up as to whether patients underwent resection or not and the subsequent outcomes. It would be prudent to also investigate other markers with this research, including P53 and the novel marker HMGB1, which are possible to detect *via* IHC analysis and therefore can be appropriately applied to clinical practice (9, 27). The thesis conducted by Alastal (15) has highlighted that the overexpression of cyclin D1 is associated with cancer presence, and that combining cyclin D1 with P53 as a panel may have a role in predicting HGD or OAC, with a specificity of 93% but a sensitivity of only 65%.

Our data also demonstrated that β-catenin was significantly overexpressed compared with the other seven markers tested, as seen in **Figure 1**. Although β-catenin did not appear to differentiate dysplasia from metaplasia, its high level of expression may prove to be significant. The signaling pathway associated with β-catenin greatly promotes cancer stem cell differentiation, i.e., precursors of mature cancer cells (41). Whether β-catenin does play a role in predicting disease progression is yet to be determined, with a wider range of participants required to fully evaluate its significance. Overexpression of β-catenin could also play a role in the identification of disease; however, this still requires endoscopic biopsy and tissue sampling for IHC, which can confirm the presence of BO histologically.

Our data have incidentally noted that Sox2 could have a role as a diagnostic marker, noting that expression was greater in metaplastic BO cells than in normal esophageal squamous cells within the same tissue sample, as shown in **Figure 3**. However, these were not control tissue samples and were part of the main BO testing tissue samples, and therefore we are unable to comment on the clinical significance of this. Sox2 is associated with an overall poorer prognosis in cancer and promotes its proliferation (42). There is also emerging evidence for the use of Sox2 as a biomarker for colorectal cancer, and again there is further evidence that its overexpression was associated with a worse prognosis (43). With this growing evidence along with our incidental findings we would go as far to say Sox2 is worth investigating further to ascertain whether it has a role as a biomarker for diagnosing higher-risk BO patients from the offset and if there is prognostic value for patients with HGD and OAC.

## Conclusion

Although our study sample consisted of a small sample size and none of the patients providing endoscopic biopsy had OAC or progressed from dysplasia to OAC, we have highlighted several potential markers for further research. Our data have further highlighted markers that have already been noted within the literature for OAC and have also suggested other known cancer markers that may be novel markers for OAC. The role of cyclin D1, potentially in combination with other markers as part of a marker panel, has real clinical applications for predicting the progression of disease, as well as possible prognostic implications. Novel markers such as β-catenin and Sox2 could have potential in diagnosing high-risk BO patients from the offset and may have use as prognostic markers; however, there is currently very limited clinical evidence of this.

We would propose a larger prospective study to further investigate the biomarkers we have highlighted to better understand their clinical application and the possible composition of a biomarker panel that will predict high-risk BO patients for whom limited resources are available.

## Data availability statement

The raw data supporting the conclusions of this article will be made available by the authors, without undue reservation.

## Ethics statement

The studies involving human participants were reviewed and approved by IRAS approval; IRAS PROJECT ID 212998, REC Reference 16/WA/0262. The patients/participants provided their written informed consent to participate in this study.

## Author contributions

LT: Data curation, writing—original draft, conceptualization, investigation, formal analysis. HA: Data curation, conceptualization. AR: Supervision, project administration, conceptualization, writing—review and editing. All authors contributed to the article and approved the submitted version.
